# Supplementary material for: Transient and general synthesis of high-density and ultrasmall nanoparticles on two-dimensional porous carbon via coordinated carbothermal shock
Source: Nat Commun. 2023 Apr 21;14:2294. doi: 10.1038/s41467-023-38023-5 (PMC10121605; doi:10.1038/s41467-023-38023-5)
Supplement: Supplementary file 1 — Supplementary Information [file 41467_2023_38023_MOESM1_ESM.pdf]

Supporting Information

Transient and General Synthesis of High-density and Ultrasmall Nanoparticles on Two-dimensional Porous Carbon via Coordinated Carbothermal Shock

Wenhui Shi[1], Zezhou Li[2], Zhihao Gong[3], Zihui Liang[1], Hanwen Liu[1], Ye-Chuang Han[4], Huiting Niu[5], Bo Song[1], Jihan Zhou[2], Xiaodong Chi[1], Hua Wang[3], Bao Yu Xia[5,*], Yonggang Yao[1,*], Zhong-Qun Tian[4,*]

[1]State Key Laboratory of Materials Processing and Die & Mould Technology, School of Materials Science and Engineering, Huazhong University of Science and Technology, Wuhan, 430074 China
[2]Beijing National Laboratory for Molecular Sciences, College of Chemistry and Molecular Engineering, Peking University, Beijing, China
[3]ZJU-Hangzhou Global Scientific and Technological Innovation Center, School of Micro-Nano Electronics, Zhejiang University, Hangzhou 311200, China
[4]State Key Laboratory of Physical Chemistry of Solid Surfaces, College of Chemistry and Chemical Engineering, Innovation Laboratory for Sciences and Technologies of Energy Materials of Fujian Province (IKKEM), Xiamen University, Xiamen 361005, China
[5]Key Laboratory of Material Chemistry for Energy Conversion and Storage (Ministry of Education), Hubei Key Laboratory of Material Chemistry and Service Failure, School of Chemistry and Chemical Engineering, Huazhong University of Science and Technology, Wuhan, 430074 Hubei, China

*E-mail: byxia@hust.edu.cn; yaoyg@hust.edu.cn; zqtian@xmu.edu.cn

This file includes:

Supplementary Figures 1 to **49**

Supplementary Tables 1 to **8**

Supplementary References

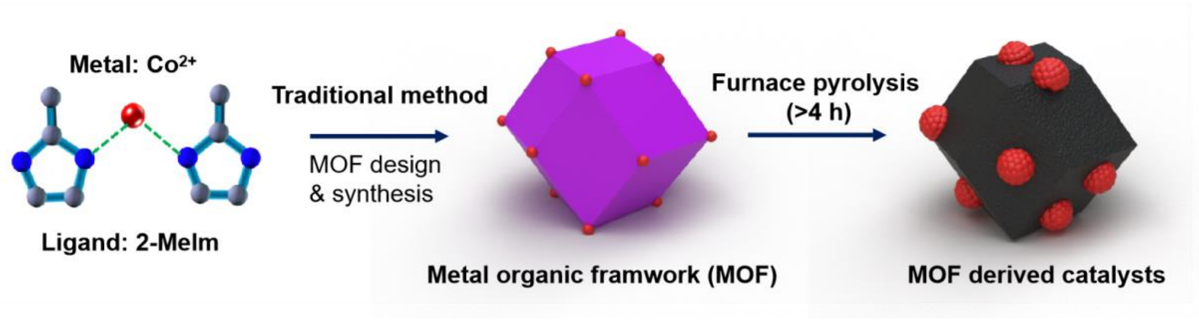

Supplementary Figure 1. MOF-derived catalysts by the traditional method, where both MOF synthesis and thermal pyrolysis take a lot of time and energy. Moreover, furnace pyrolysis leads to particle aggregation and buried in the 3D carbon matrix.

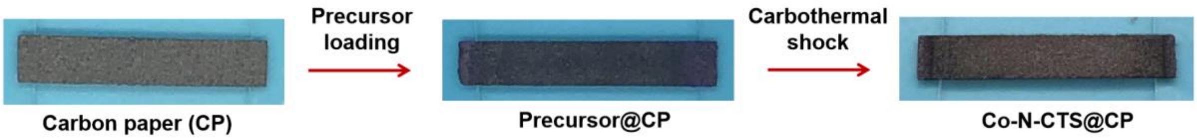

Supplementary Figure 2. The synthesis process of Co-N-CTS.

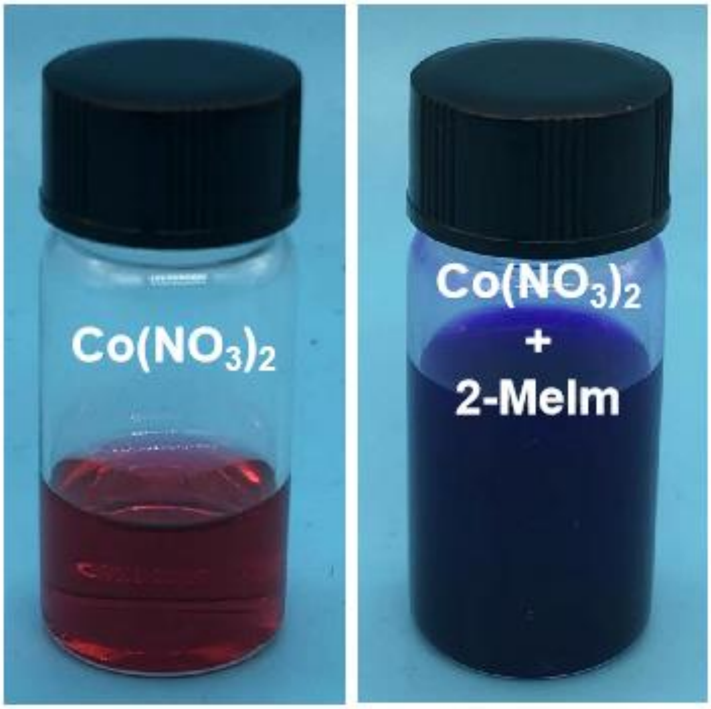

Supplementary Figure 3. Photo image of crimson $Co(NO_3)_2$ and blue mixture solution after adding 2-MeIm solution.

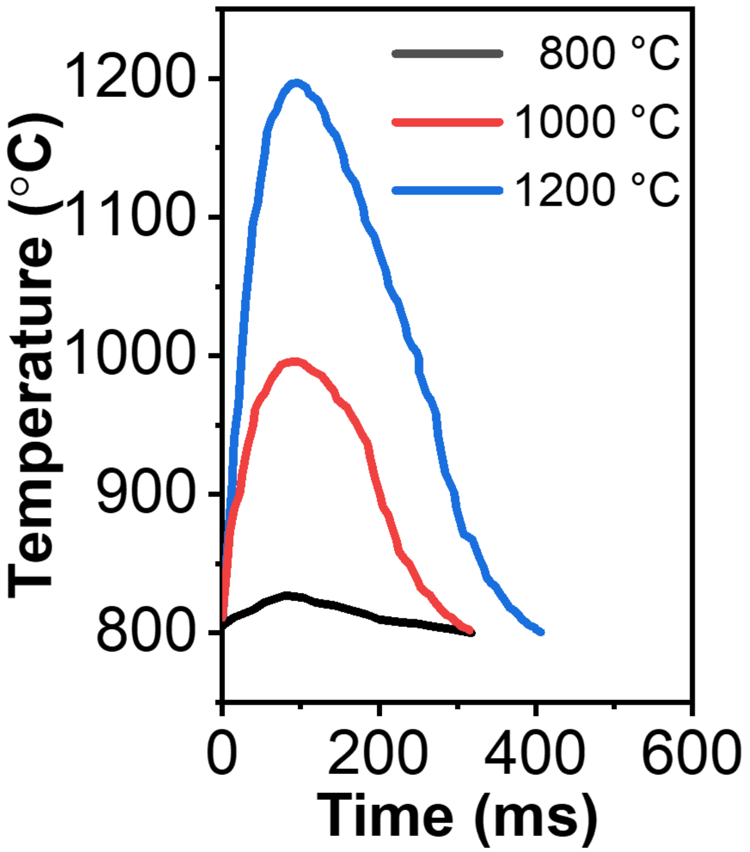

Supplementary Figure 4. The temperature profile of carbothermal shock treatment (800, 1000, and 1200 °C, 100 ms).

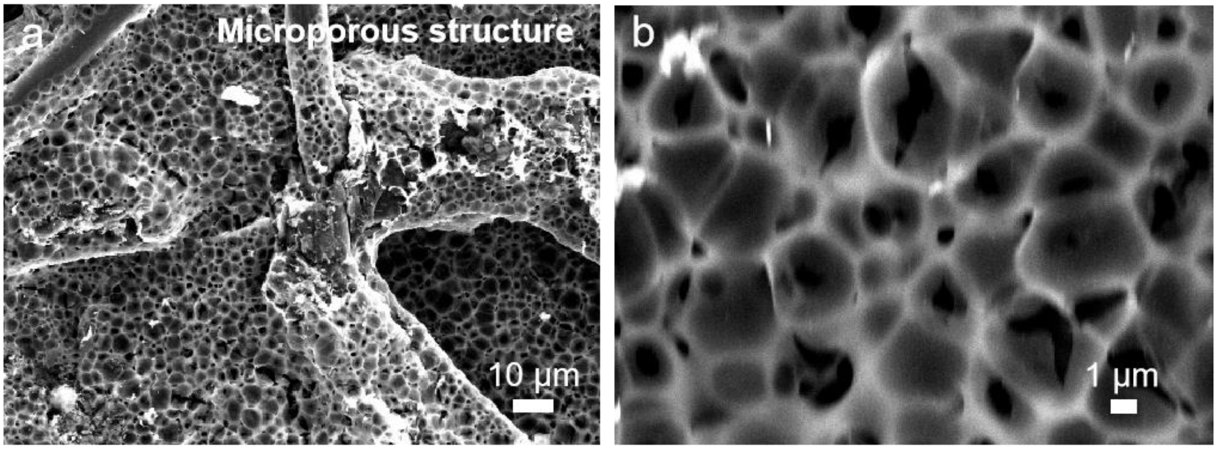

Supplementary Figure 5. SEM image of MOC after carbothermal shock.

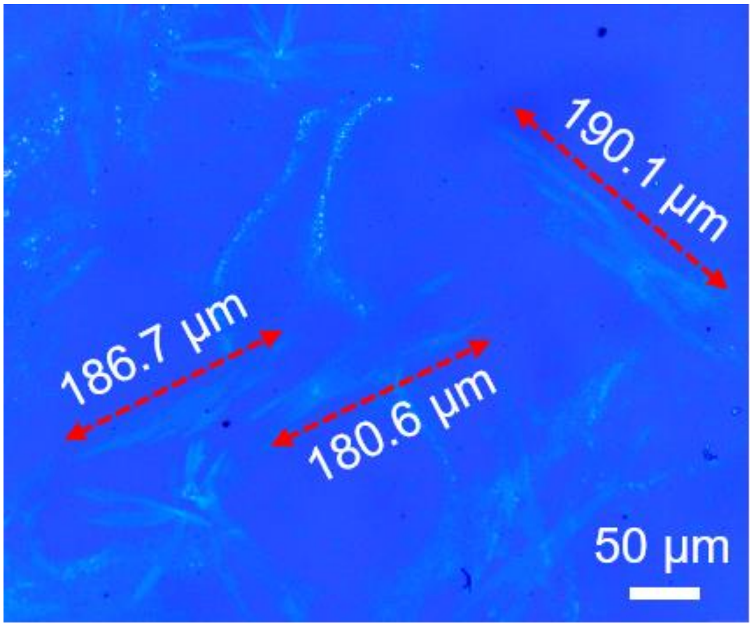

Supplementary Figure 6. The optical microscopy image of Co-N-CTS.

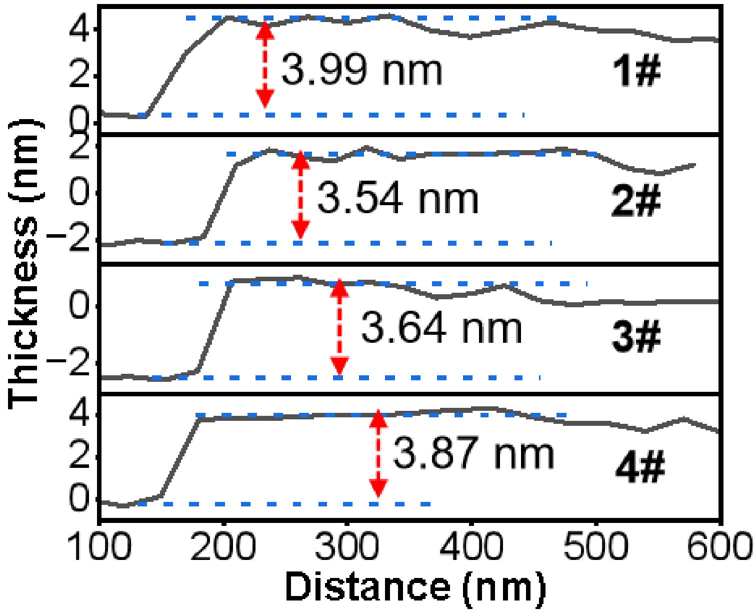

Supplementary Figure 7. The thickness of different carbon films.

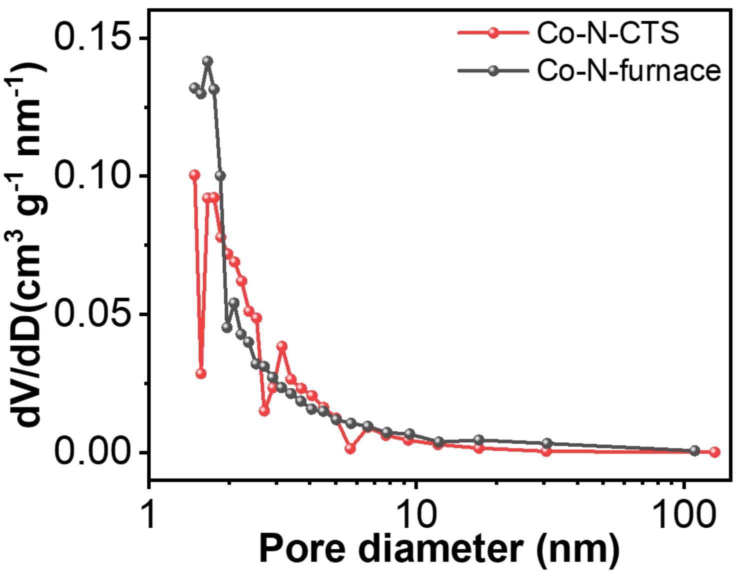

Supplementary Figure 8. The pore size distribution of Co-N-CTS and Co-N-furnace.

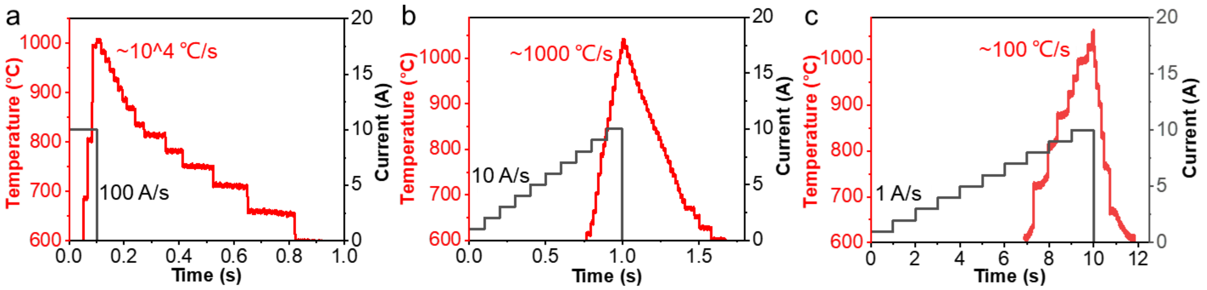

Supplementary Figure 9. The current and temperature profiles at different (designed) heating rates: (a) ~10^4 ℃/s, (b) ~1000 ℃/s, and (c) ~100 ℃/s.

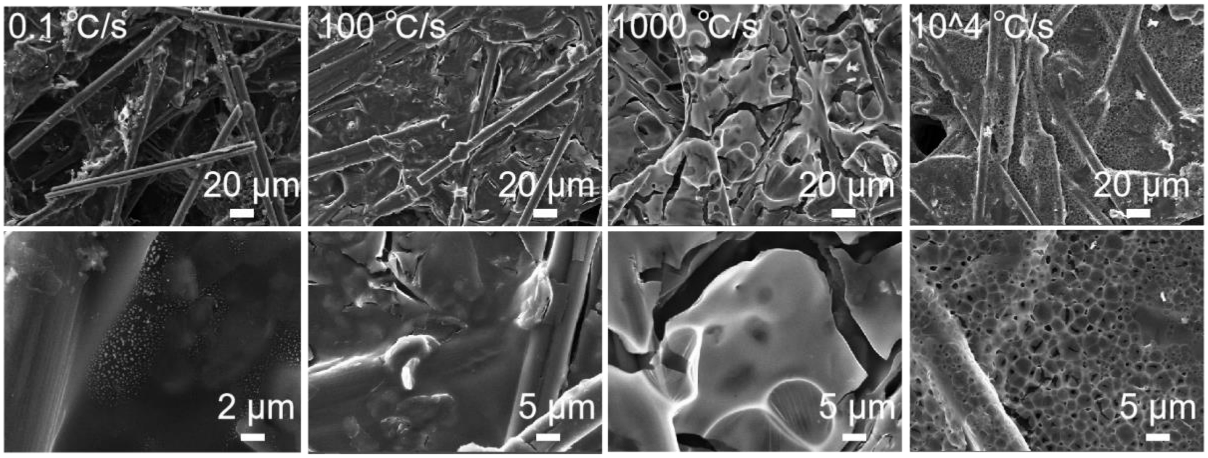

Supplementary Figure 10. SEM images of MOC at different heating rates of 0.1, 100, 1000, and 10^4 ℃/s**.**

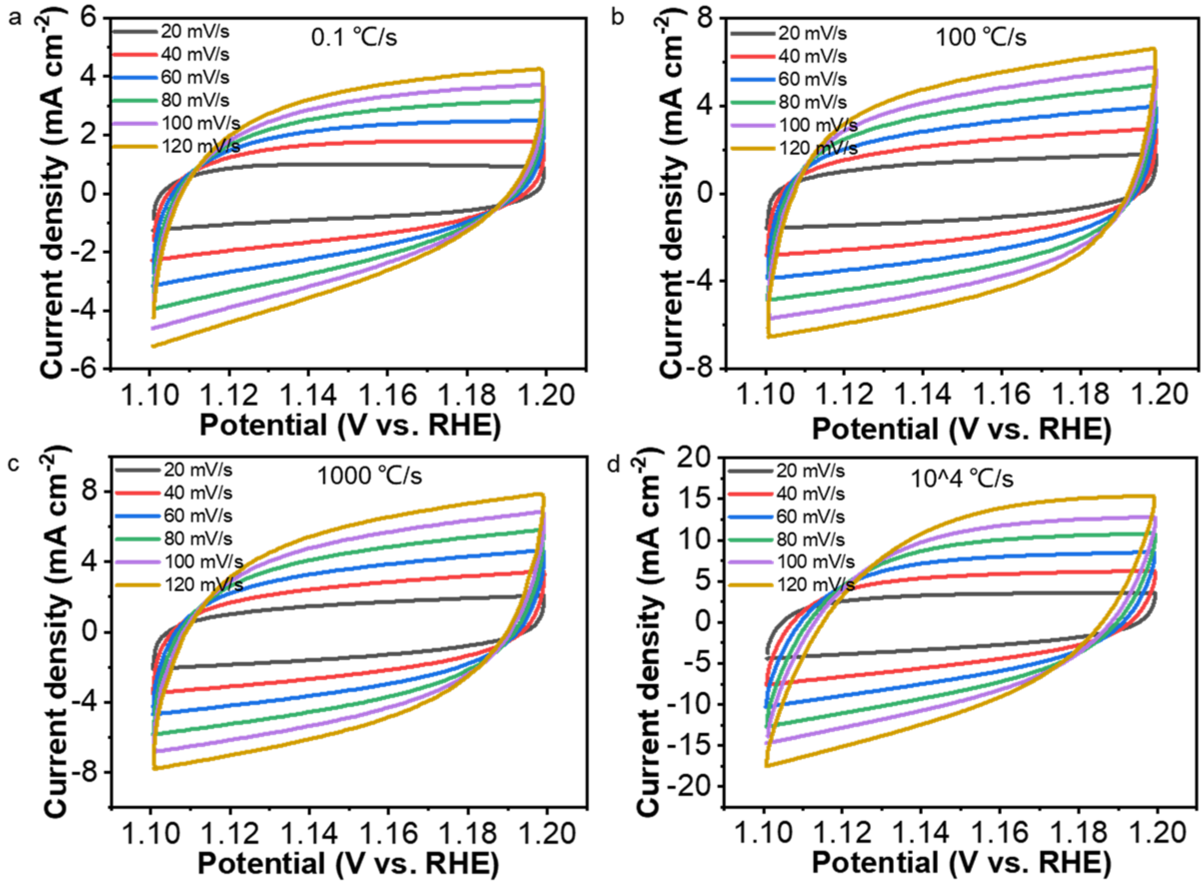

Supplementary Figure 11. CV curves of Co sample at different heating rate 0.1, 100, 1000 and 10^4 ℃/s.

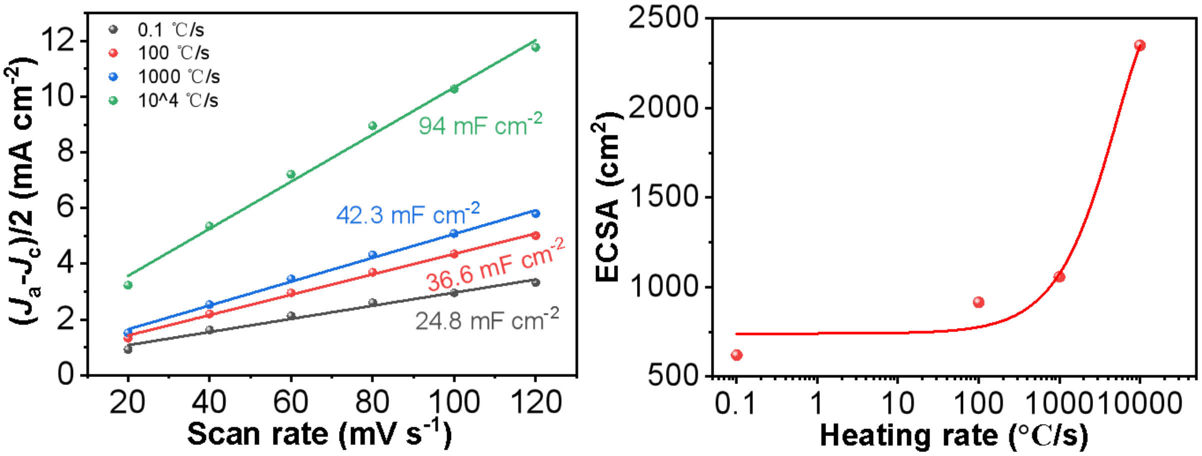

Supplementary Figure 12. (a) the C_{dl} values of Co sample at different heating rate, and (b) the relationship between ECSA and heating rate.

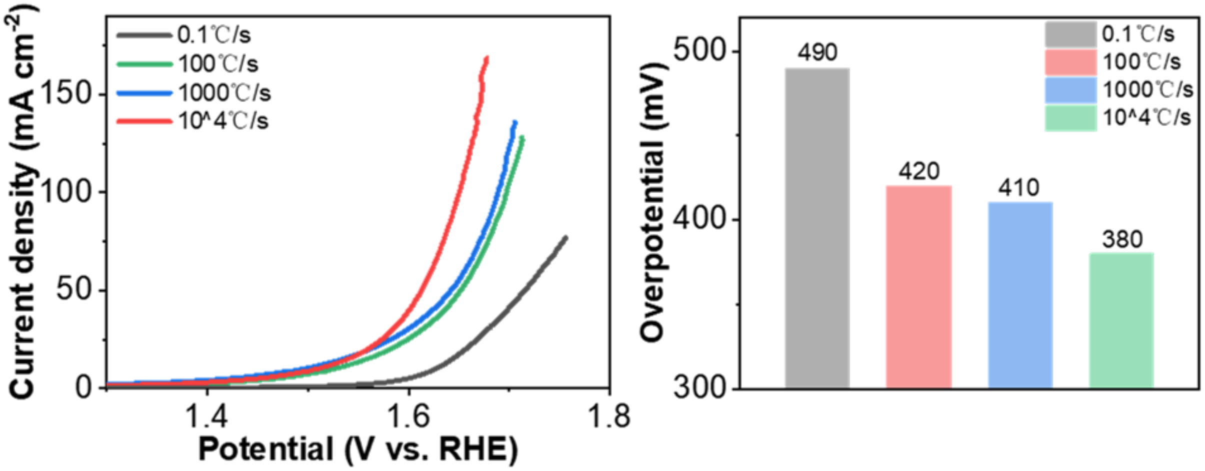

Supplementary Figure 13. The OER performances of Co samples at different heating rates of 0.1, 100, 1000, and 10^4 °C/s**.**

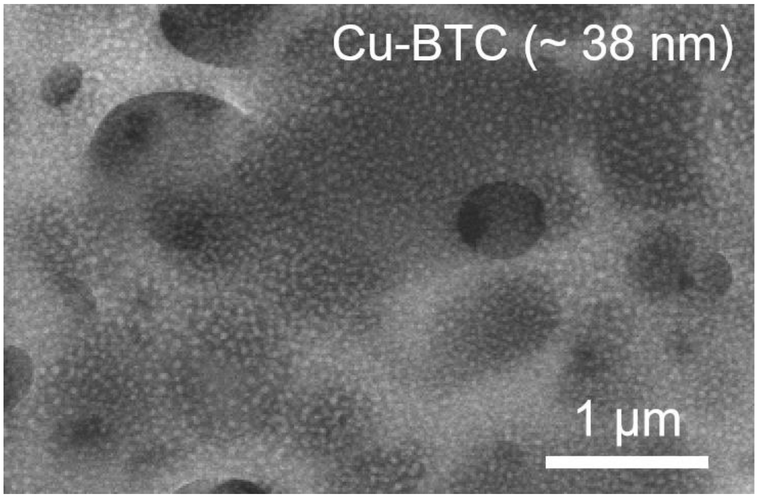

Supplementary Figure 14. SEM images of Cu-BTC (~38 nm) after CTS treatment.

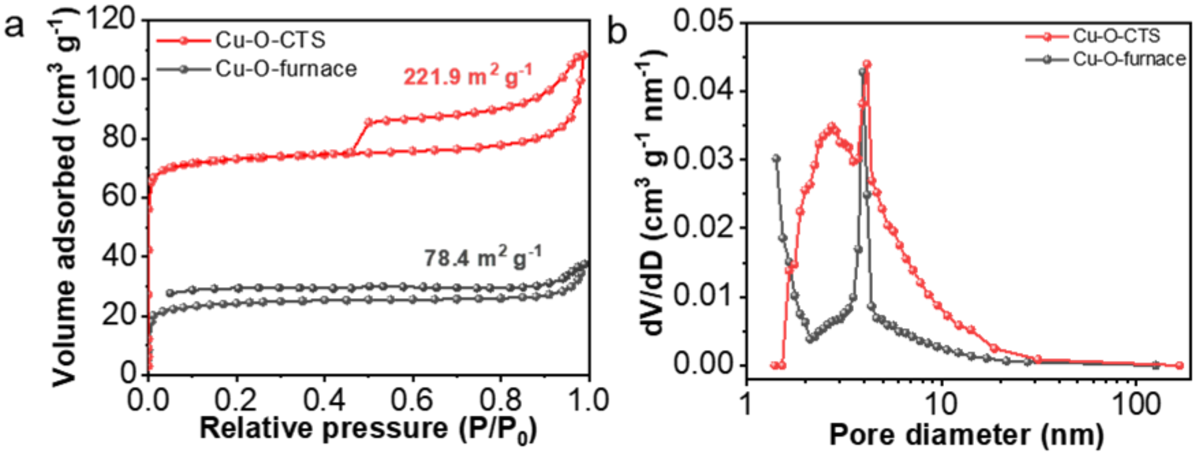

Supplementary Figure 15. the N_2 adsorption-desorption isotherms and pore size distribution of Cu-O-CTS, and Cu-O-furnace.

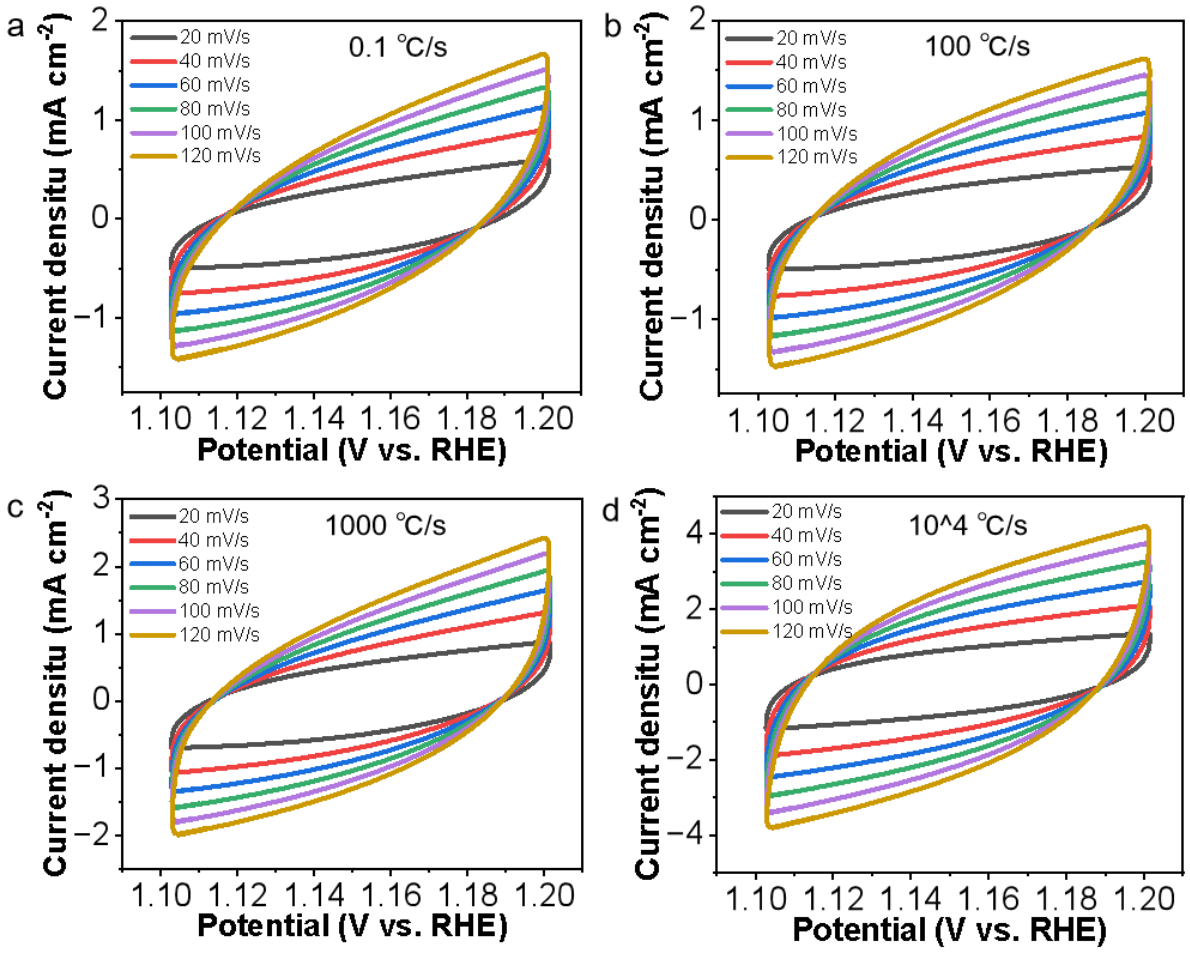

Supplementary Figure 16. The CV curves of Cu sample at different heating rate 0.1, 100, 1000 and 10^4 °C/s.

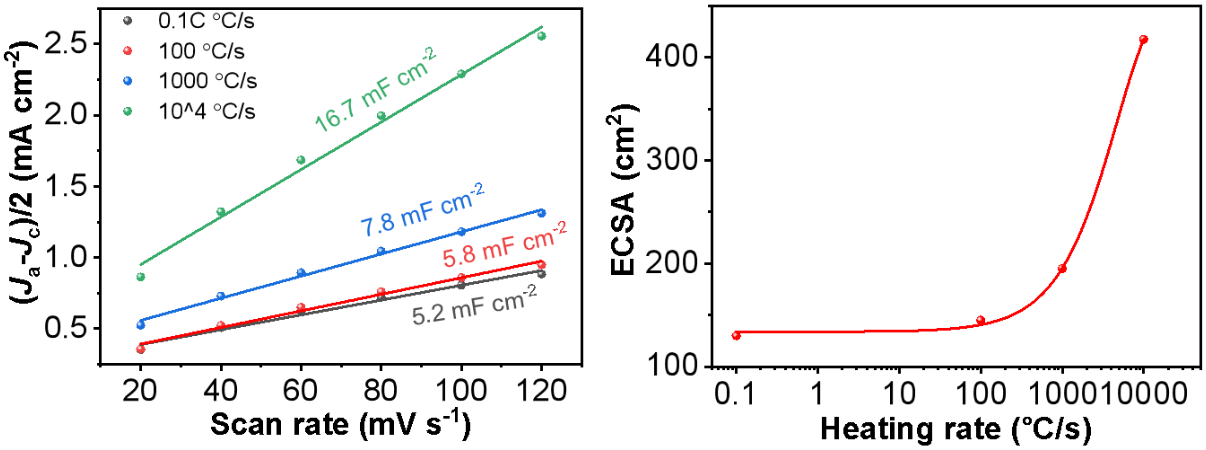

Supplementary Figure 17. (a) the C_{dl} values of Cu sample at different heating rate, and (b) the relationship between ECSA and heating rate.

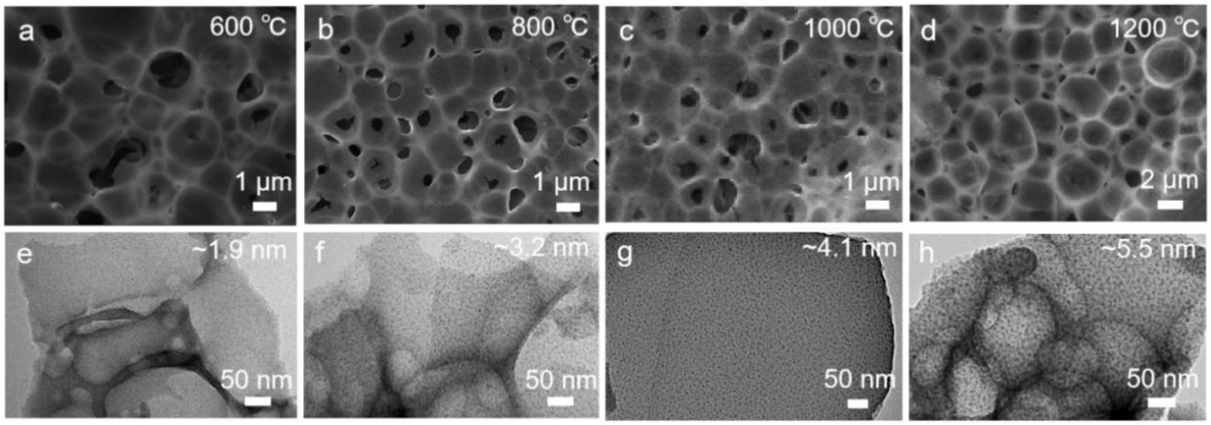

Supplementary Figure 18. (a-d) SEM and (e-f) TEM images of Co-N-CTS samples pyrolyzed at different temperatures (600, 800, 1000, and 1200 °C).

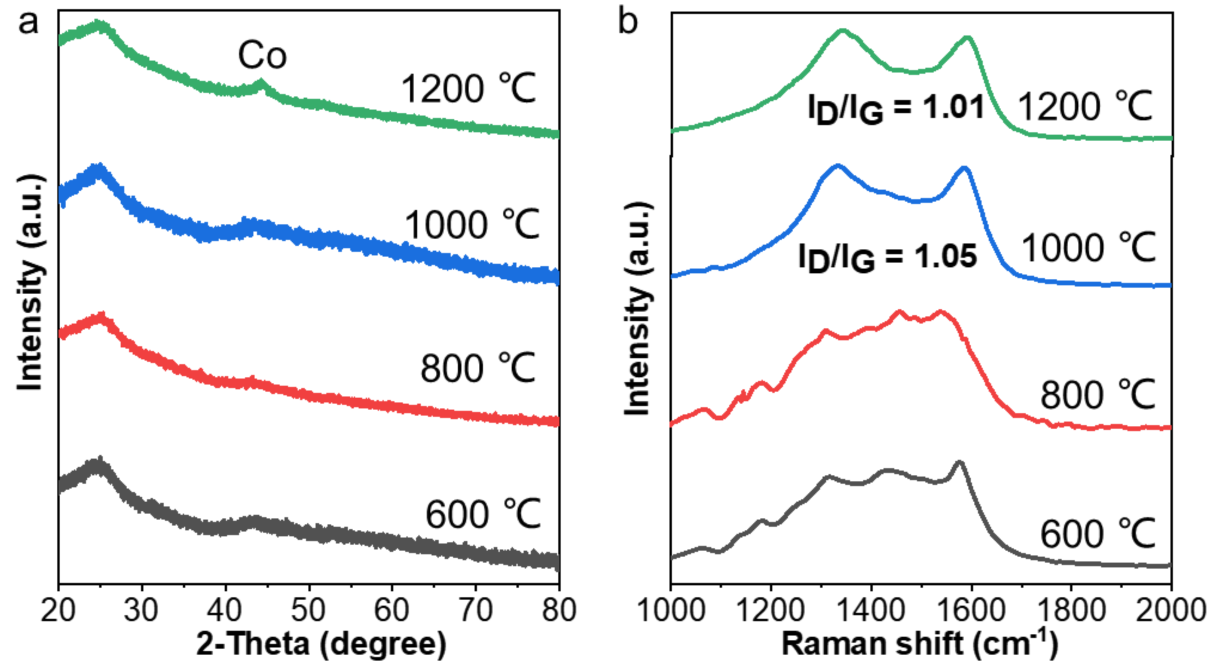

Supplementary Figure 19. (a) XRD and (b) Raman spectrum of Co-N-CTS samples pyrolyzed at different temperatures (600, 800, 1000, and 1200 °C).

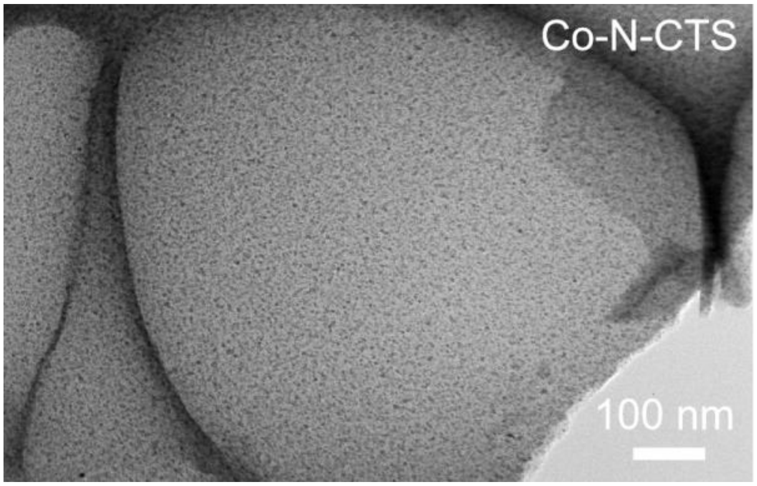

Supplementary Figure 20. TEM image of Co-N-CTS.

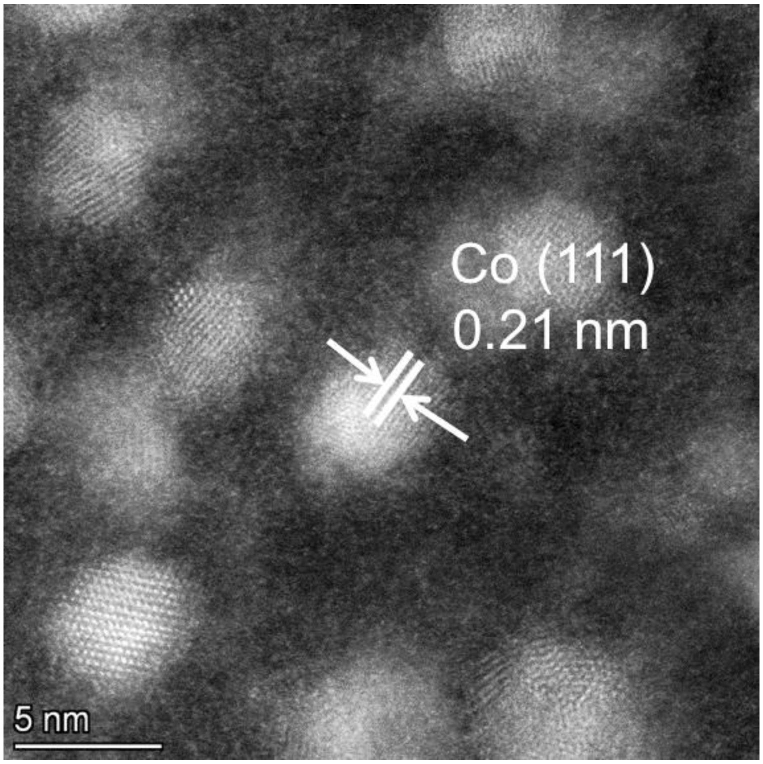

Supplementary Figure 21. High-resolution TEM image of Co-N-CTS.

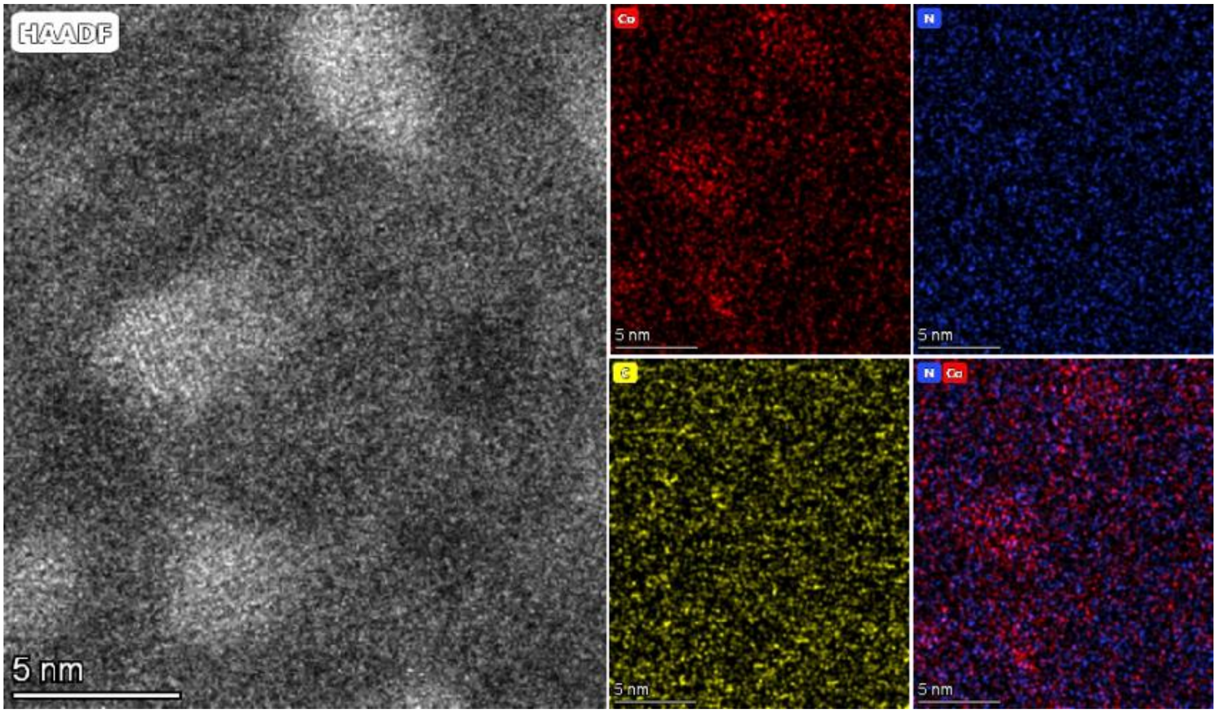

Supplementary Figure 22. High-magnification HAADF-STEM image and EDS mapping of Co-N-CTS.

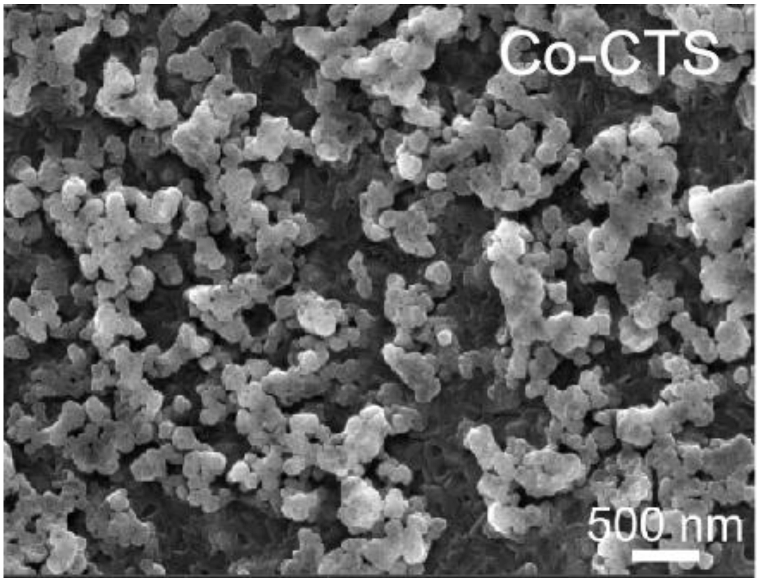

Supplementary Figure 23. SEM image of Co without 2-MeIm after carbothermal shock.

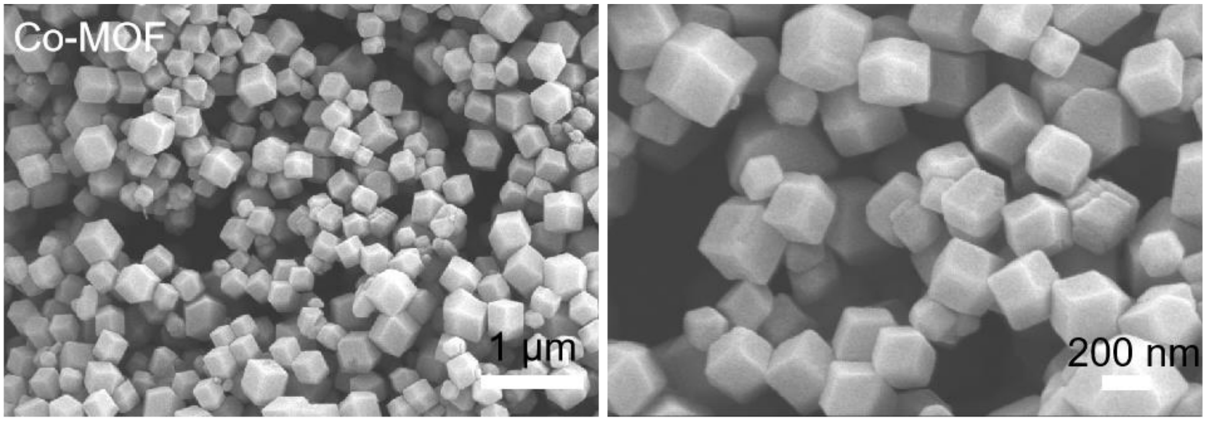

Supplementary Figure 24. SEM image of Co-MOF.

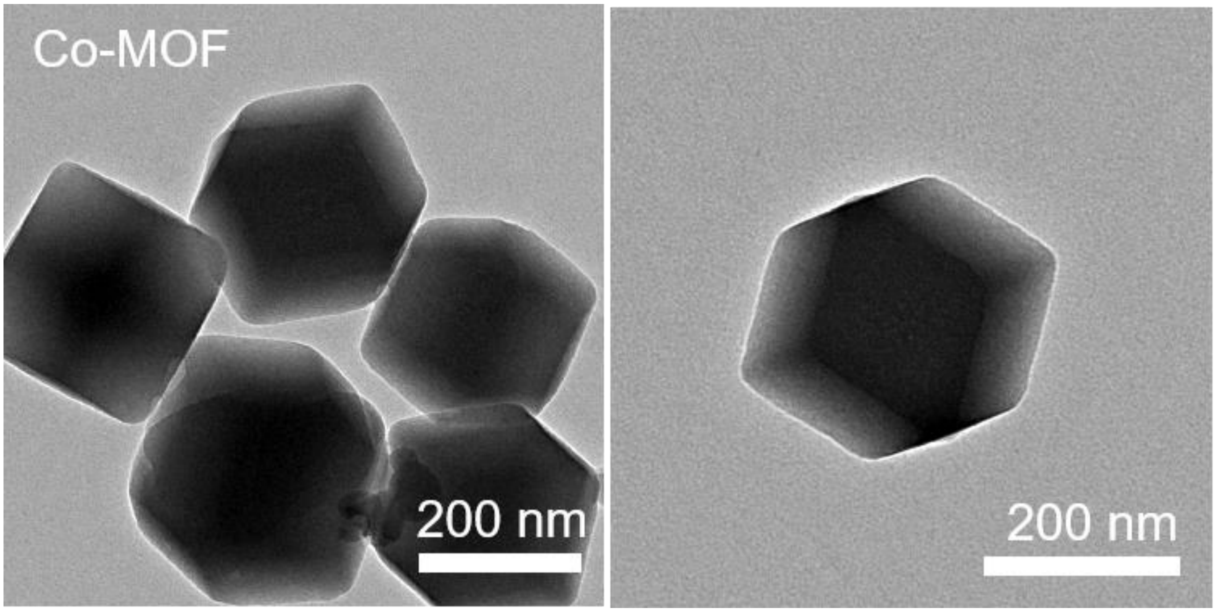

Supplementary Figure 25. TEM image of Co-MOF.

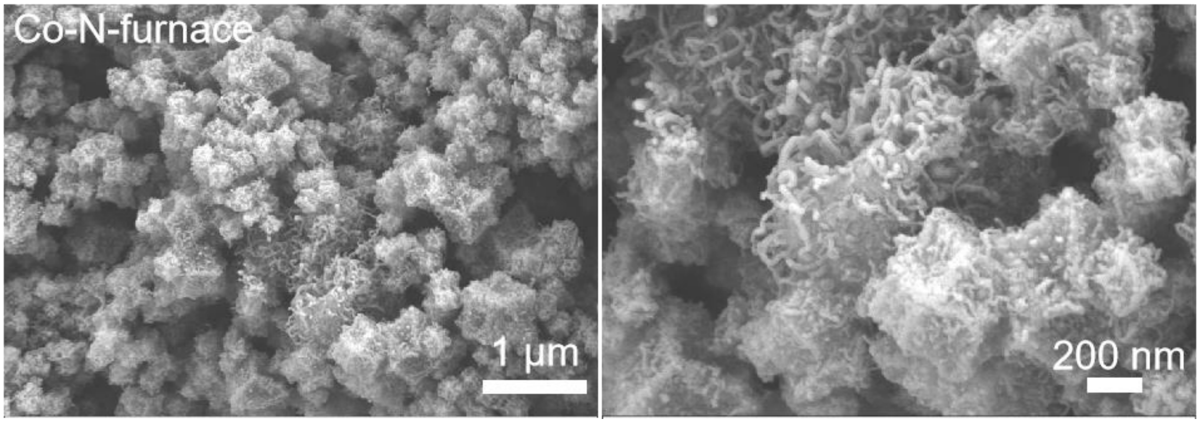

Supplementary Figure 26. SEM image of Co-MOF after furnace heating treatment.

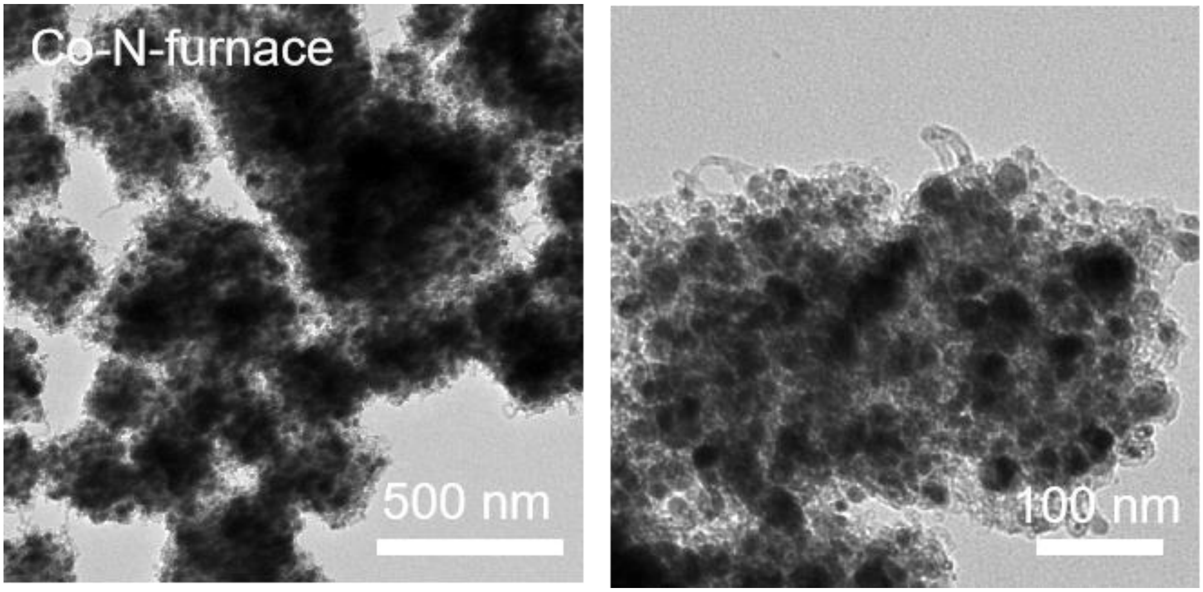

Supplementary Figure 27. TEM image of Co-MOF after furnace heating treatment.

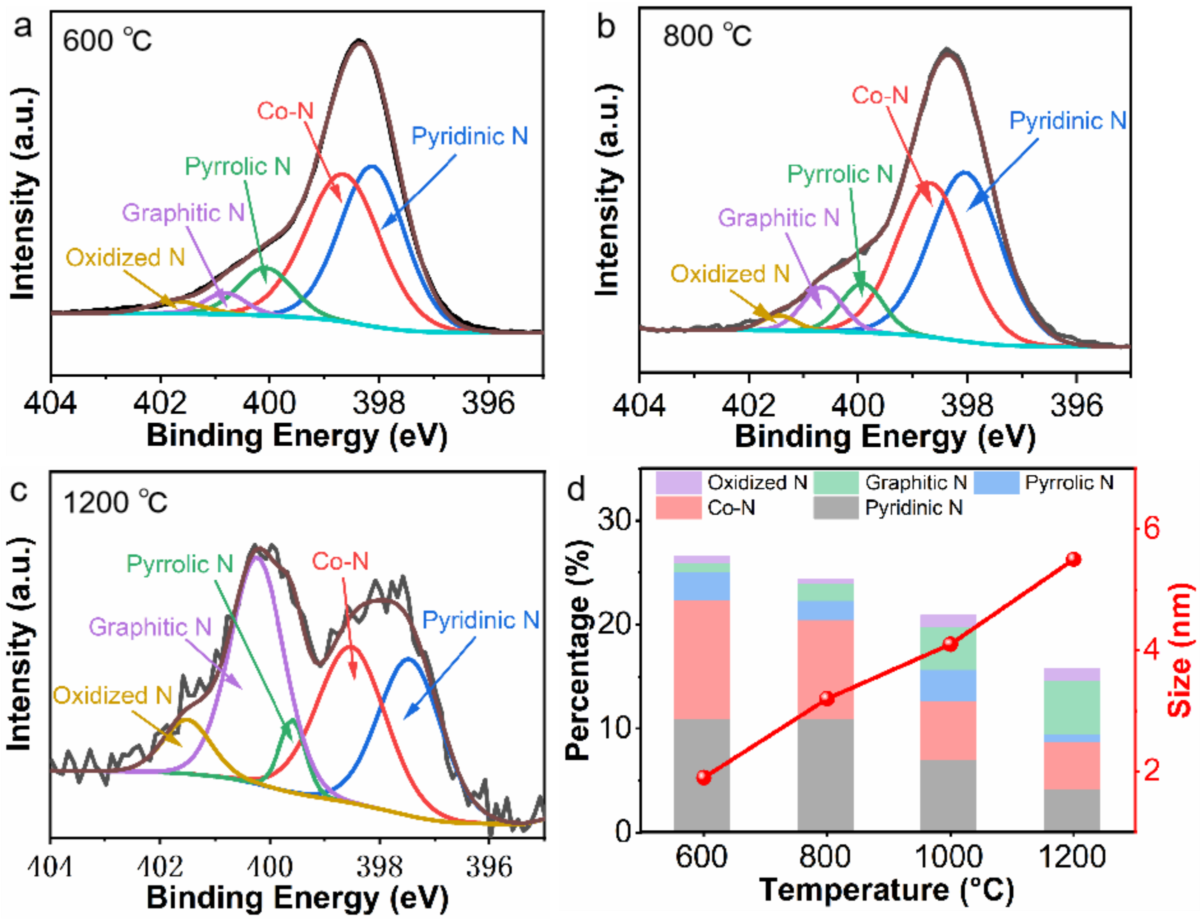

Supplementary Figure 28. The high-resolution XPS of N signal at 600, 800, and 1200 °C and the contents of N species and particle size pyrolyzed at different temperatures.

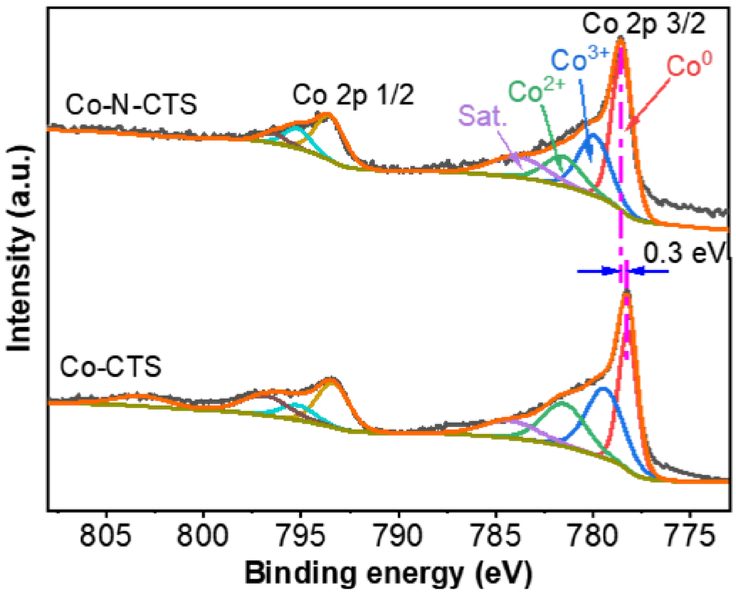

Supplementary Figure 29. The XPS spectrum of Co-N-CTS and Co-CTS.

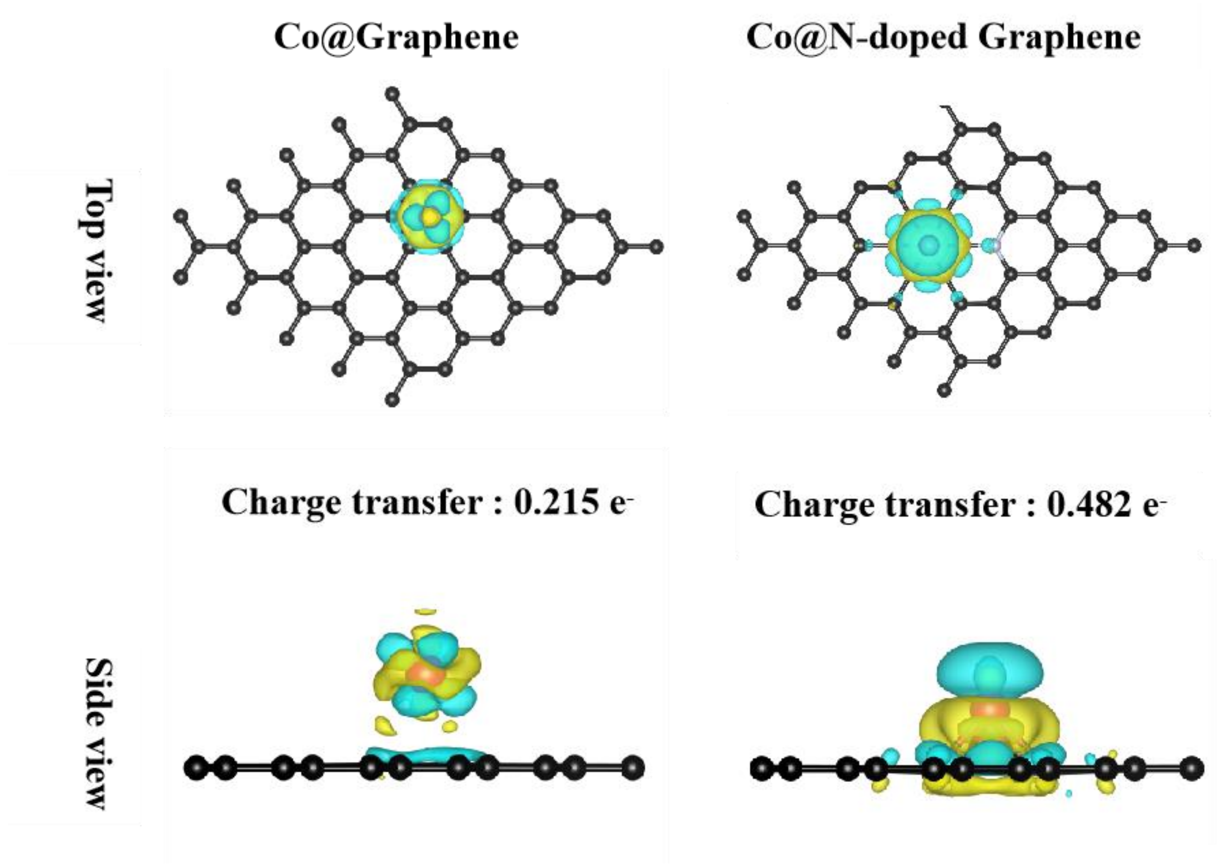

Supplementary Figure 30. The difference of charge density between Co and the rest of the system. Yellow represents the electron-accumulation area and cyan is the electron-depletion area. Isosurface level: 0.002 e/Bohr^3.

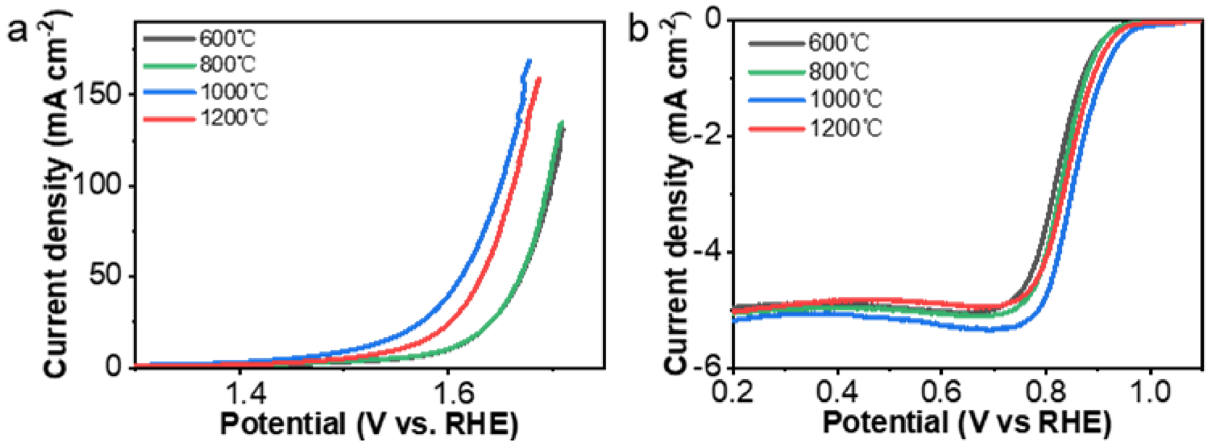

Supplementary Figure 31. (a) OER and (b) ORR performances of Co-N-CTS samples at different pyrolysis temperature of 600, 800, 1000, and 1200 °C.

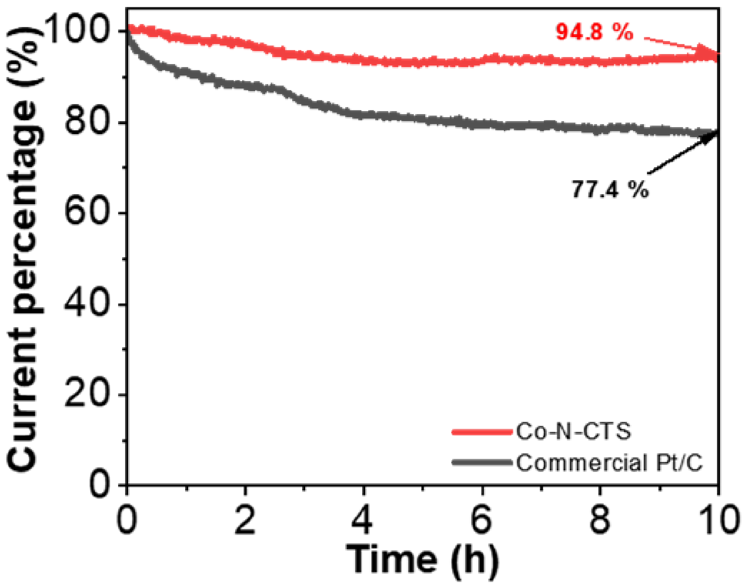

Supplementary Figure 32. The ORR stability tests of commercial Pt/C and Co-N-CTS.

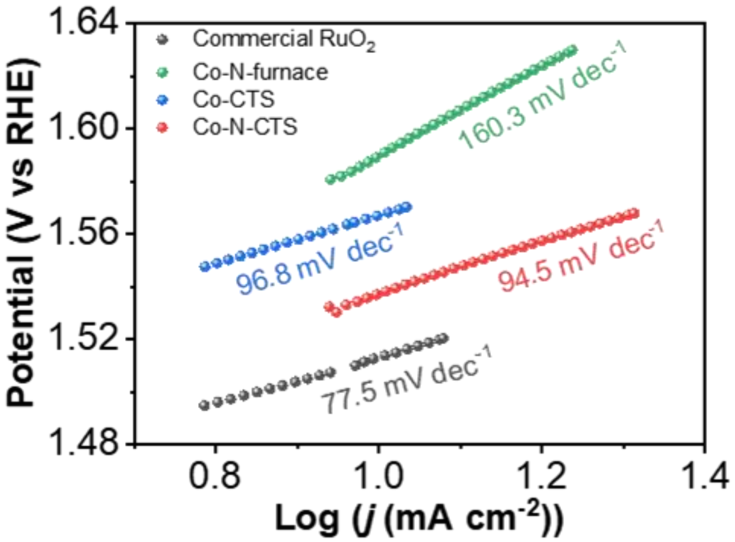

Supplementary Figure 33. The Tafel slope of commercial RuO_2, Co-N-furnace, Co-CTS, and Co-N-CTS.

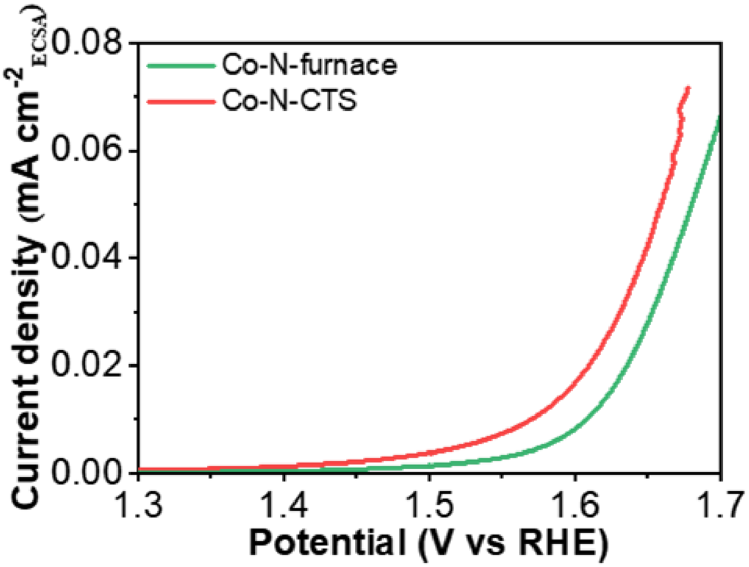

Supplementary Figure 34. The ECSA-normalized LSV curves of Co-N-CTS and Co-N-furnace.

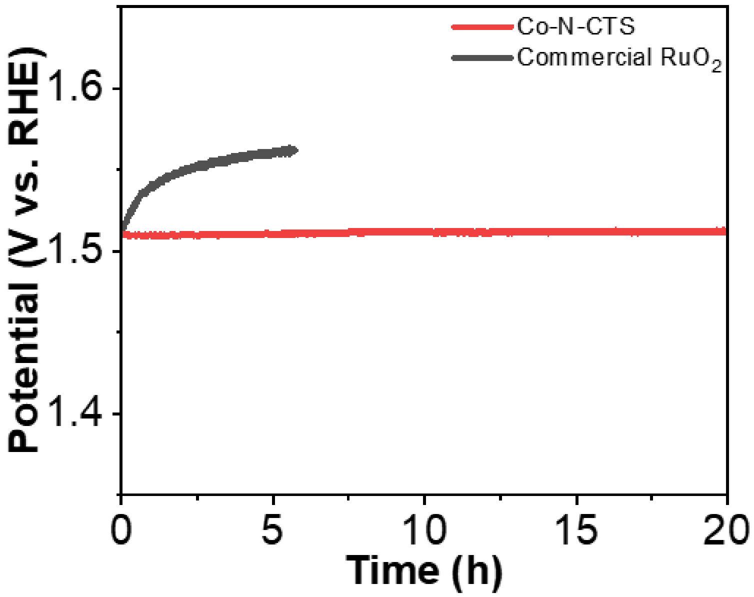

Supplementary Figure 35. The OER stability tests of commercial RuO_2 and Co-N-CTS.

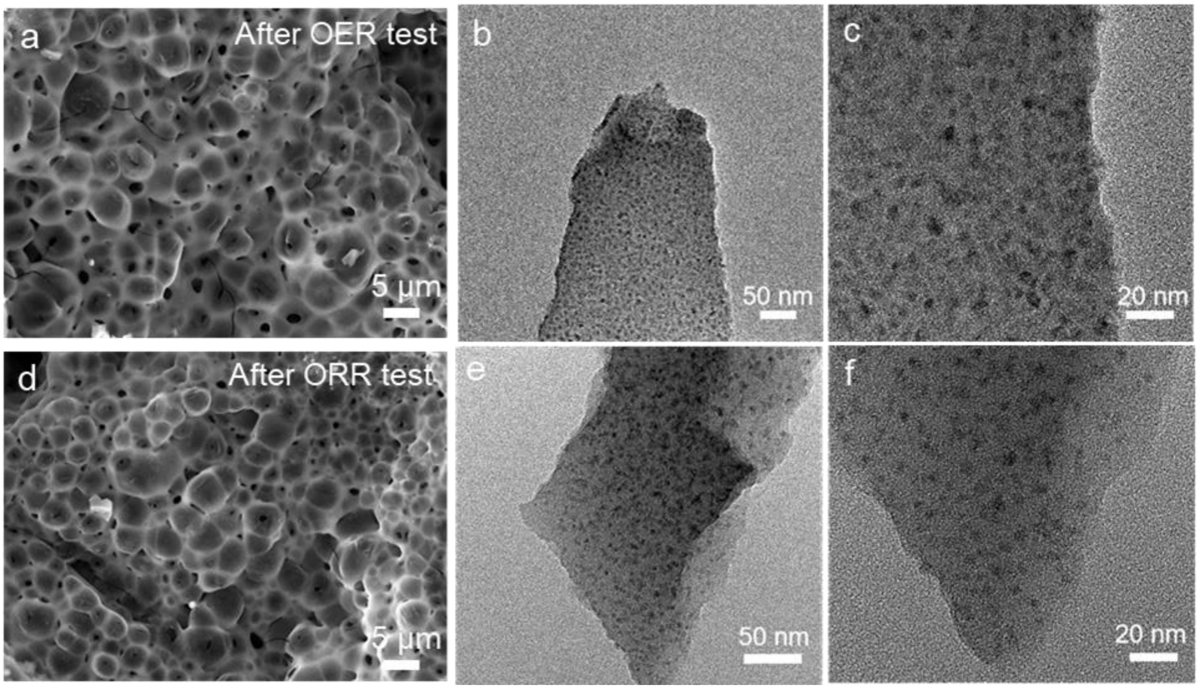

Supplementary Figure 36. SEM and TEM image of Co-N-CTS after 20 h OER and 10 h ORR test.

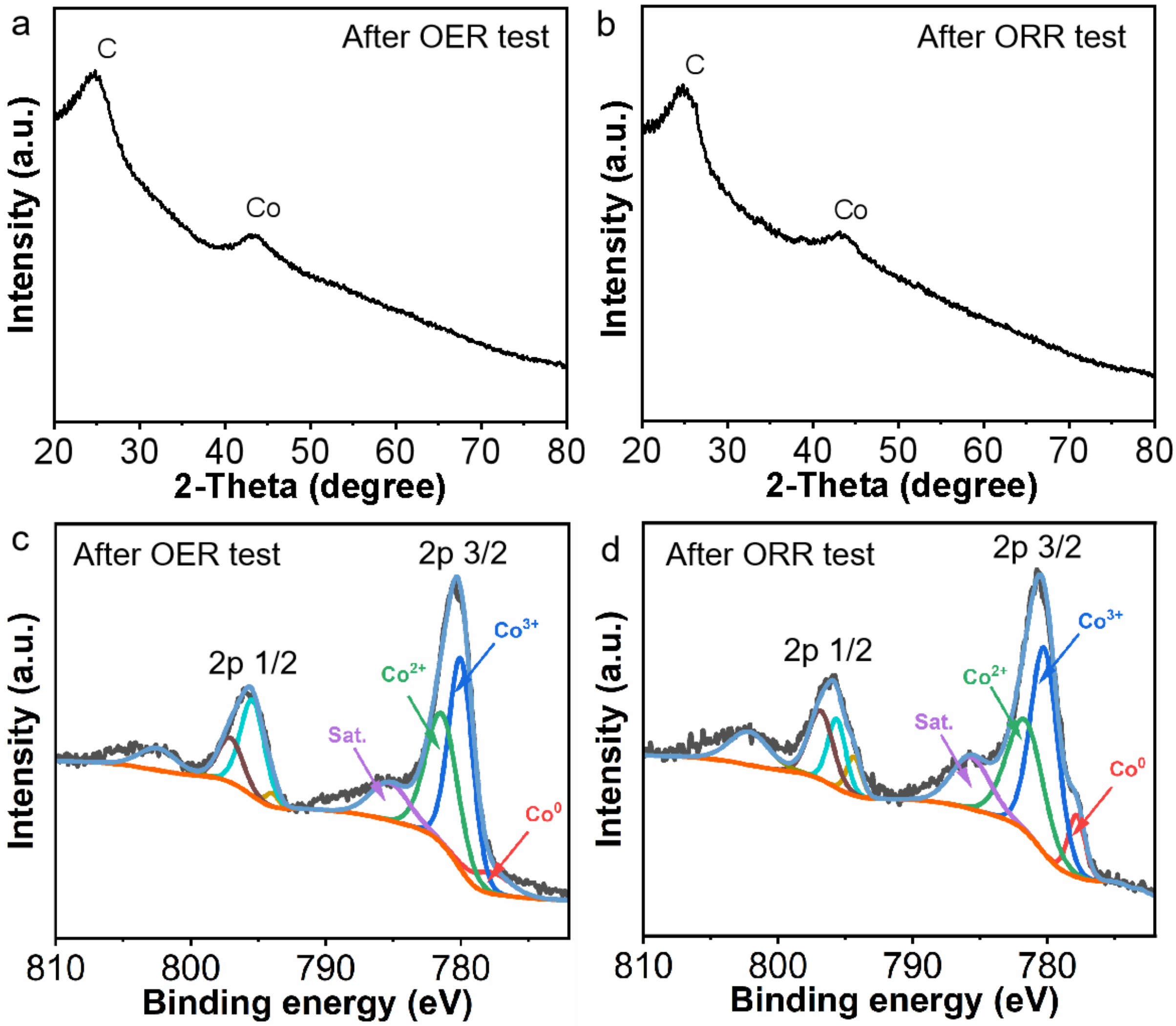

Supplementary Figure 37. The XRD and Co XPS spectrum of Co-N-CTS after 20 h OER and 10 h ORR test.

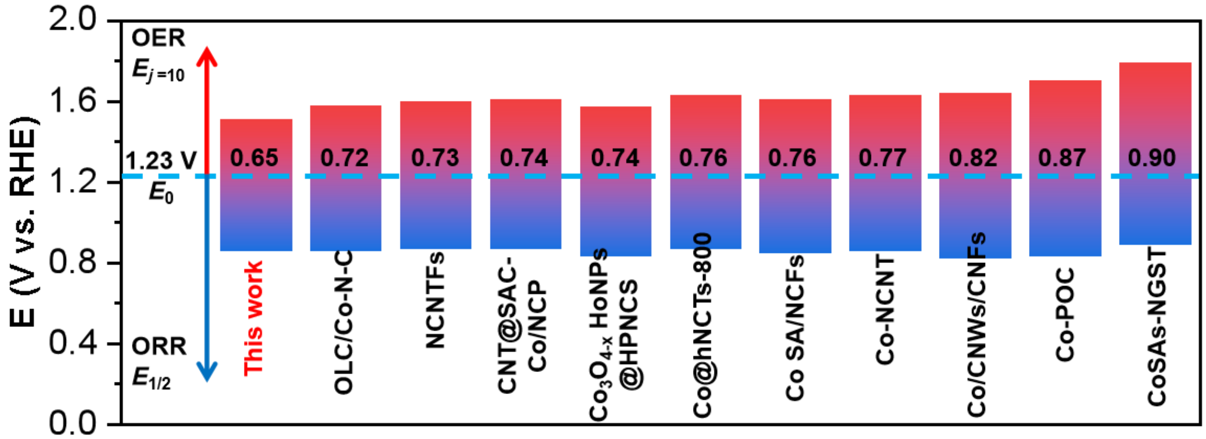

Supplementary Figure 38. Comparison of the bifunctional oxygen electrocatalytic performances ($E_{1/2}$ in ORR and $E_{j=10}$ in OER, the marked data is the potential difference between OER ($E_{j=10}$) and ORR ($E_{1/2}$) ($\Delta E = E_{j=10} - E_{1/2}$)) between Co-N-CTS and other reported Co-based bifunctional electrocatalysts in the literature.

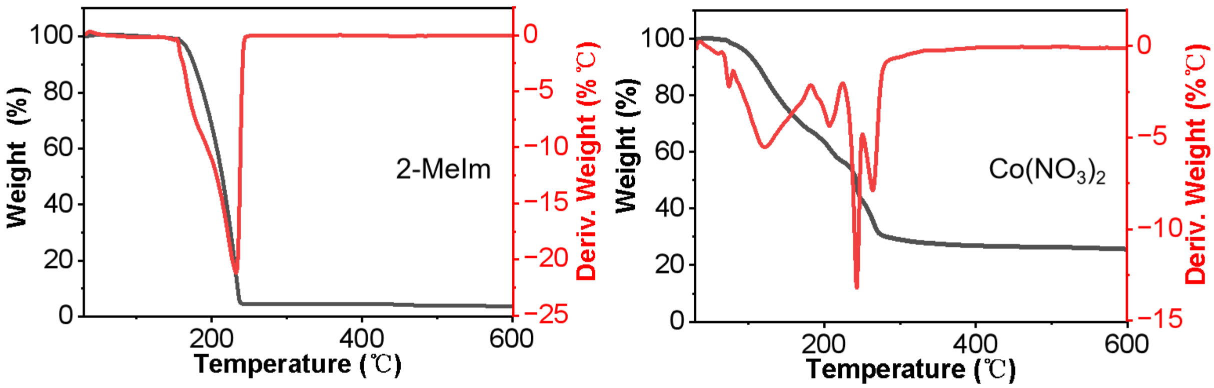

Supplementary Figure 39. The TGA-DTG profile of (a) 2-MeIm and (b) $Co(NO_3)_2$ from 50 to 600 °C in Ar gas.

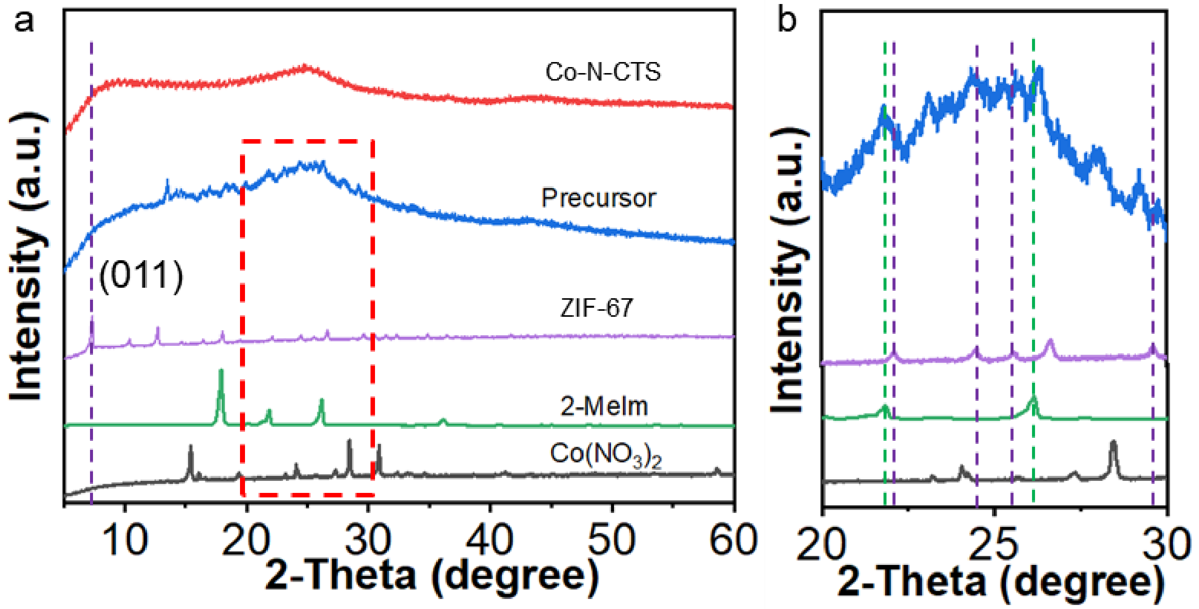

Supplementary Figure 40. (a) and (b) XRD patterns of the metal-ligand precursor, Co-N-CTS, ZIF-67, 2-MeIm, and $Co(NO_3)_2$.

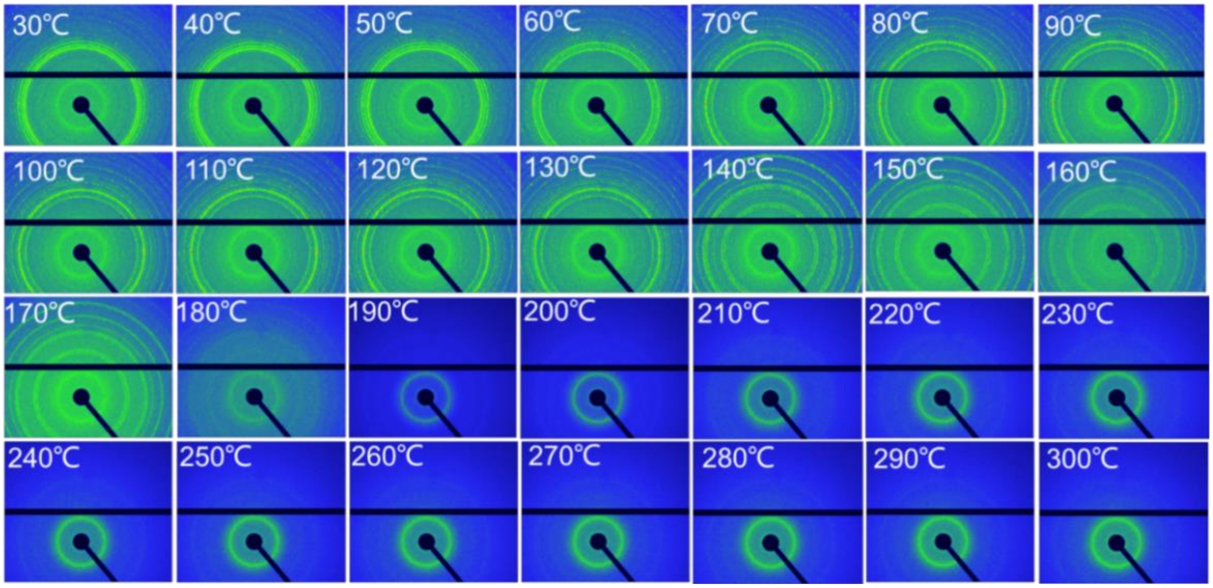

Supplementary Figure 41. The diffraction pattern of MOC from 30 to 300 ℃.

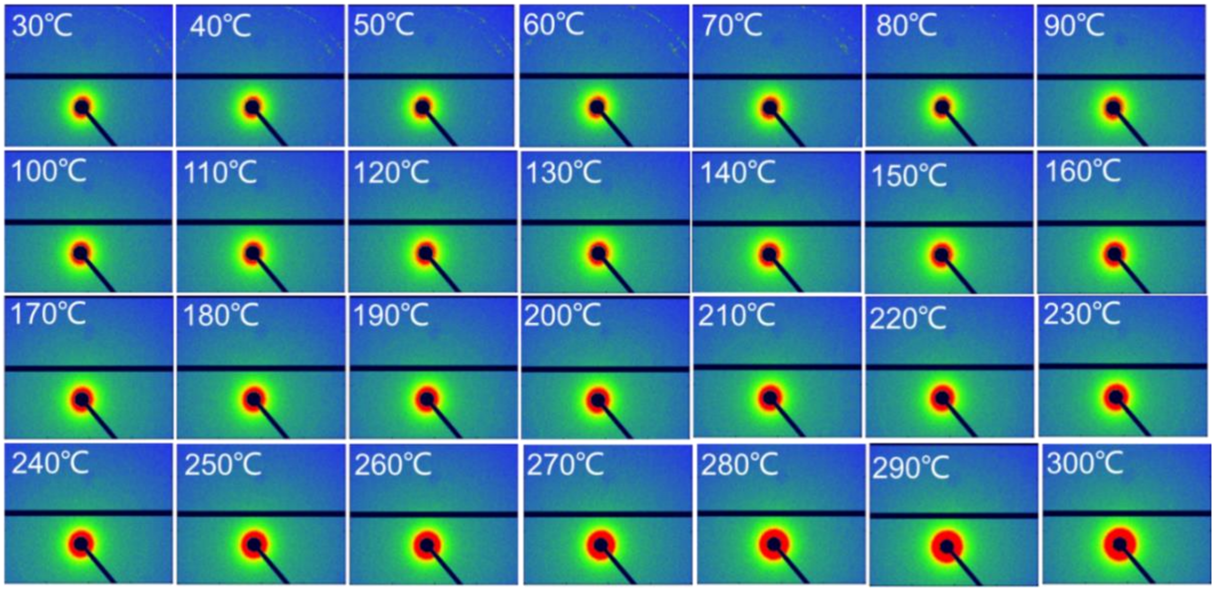

Supplementary Figure 42. The diffraction pattern of Cu-2-MeIm from 30 to 300 °C.

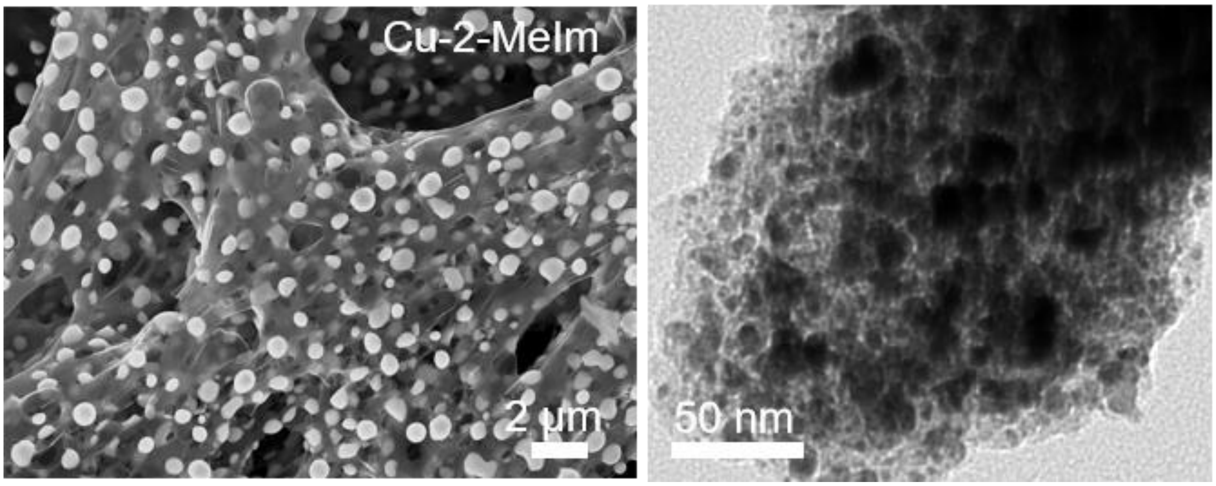

Supplementary Figure 43. SEM and TEM image of Cu-2-MeIm after carbothermal shock.

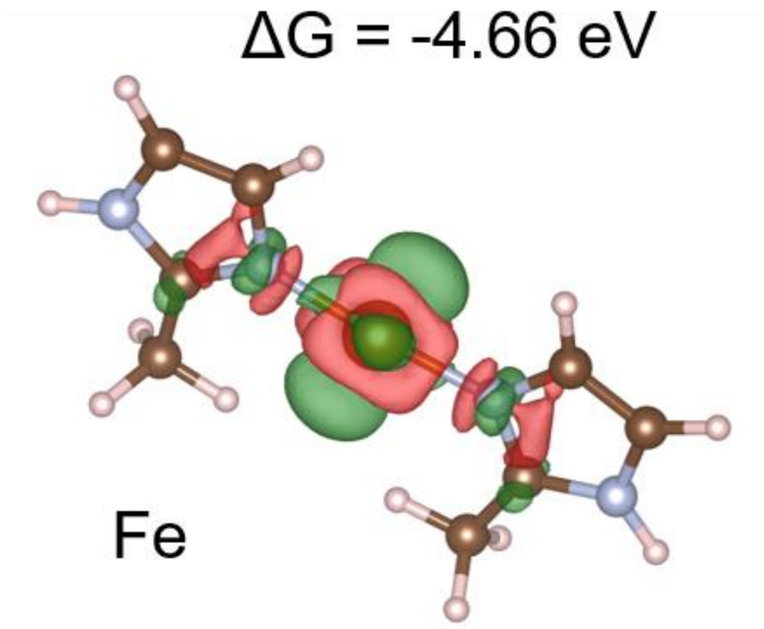

Supplementary Figure 44. The formation energy of Fe with 2-MeIm.

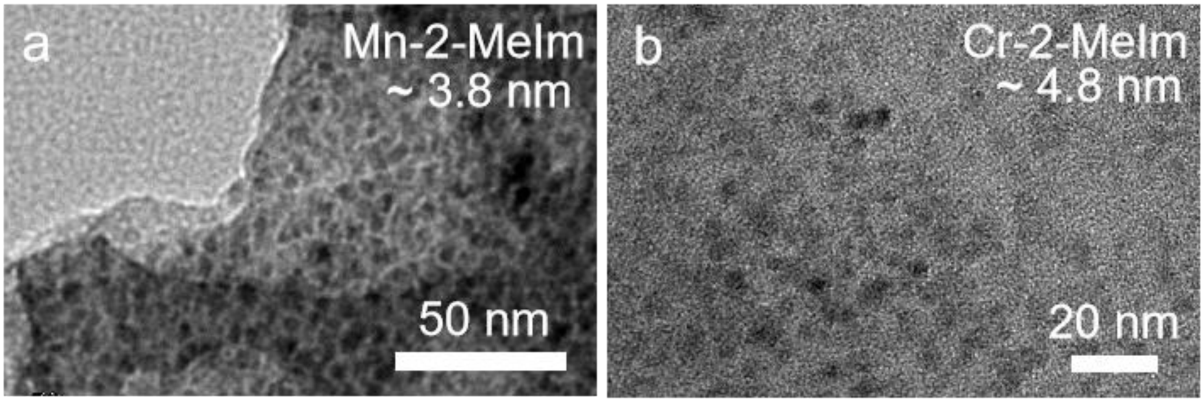

Supplementary Figure 45. TEM image of (a) Mn-2-MeIm and (b) Cr-2-MeIm after carbothermal shock.

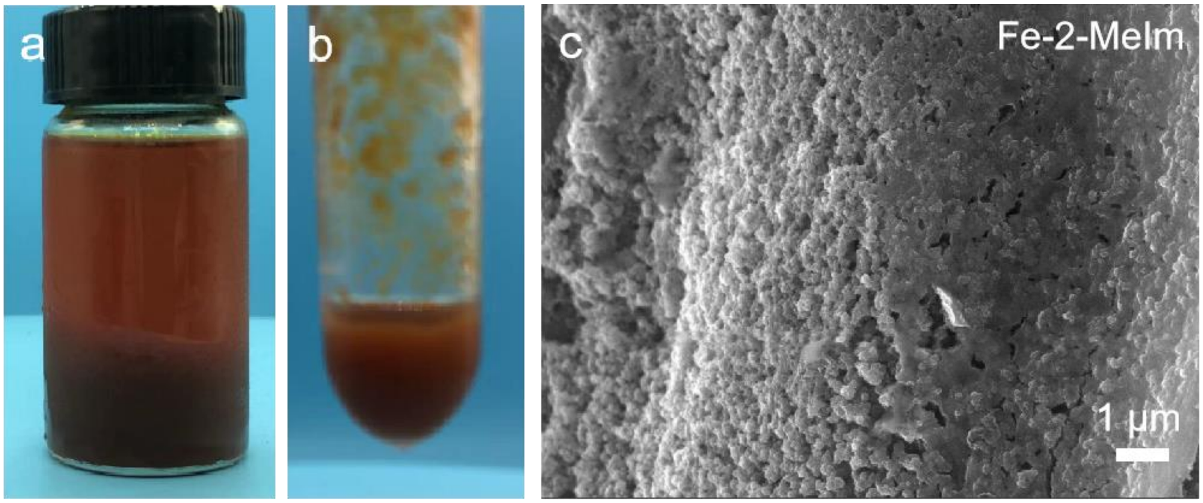

Supplementary Figure 46. Optical image of (a) 0.2 M $Fe(NO_3)_3$ ethanol solution, (b) $Fe(NO_3)_3$ and 2-MeIm mixed solution, and (c) SEM image of Fe-2-MeIm after carbothermal shock. The $Fe(OH)_3$ precipitated in 0.2 M $Fe(NO_3)_3$ ethanol solution after 2 h, and the $Fe(OH)_3$ nanoparticles precipitated immediately after the $Fe(NO_3)_3$ solution was added to the 2-MeIm solution, indicating that alkaline 2-MeIm promotes $Fe(OH)_3$ nanoparticle formation.

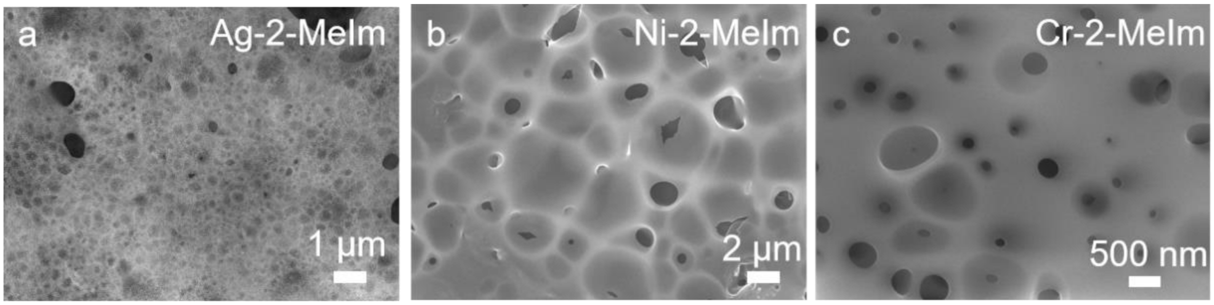

Supplementary Figure 47. SEM images of (a) Ag-2-MeIm, (b) Ni-2-MeIm, and (c) Cr-2-MeIm after carbothermal shock.

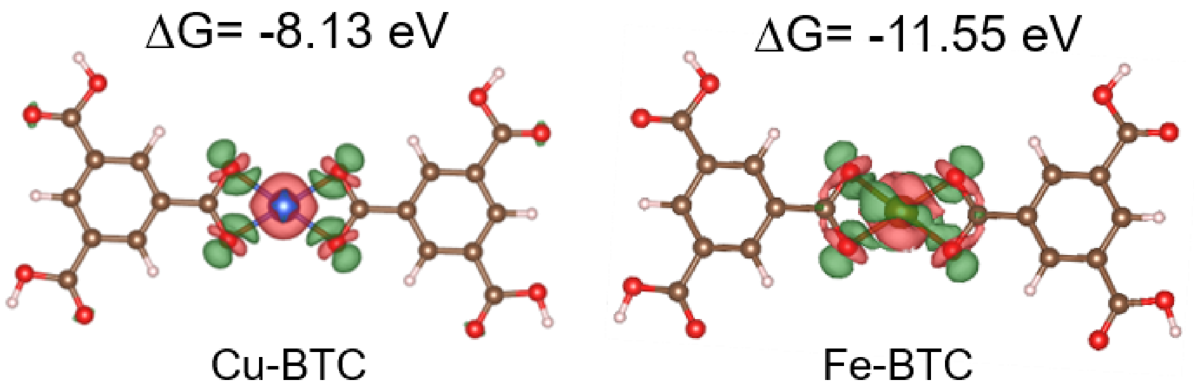

Supplementary Figure 48. The formation energy and charge distribution of Cu and Fe with BTC.

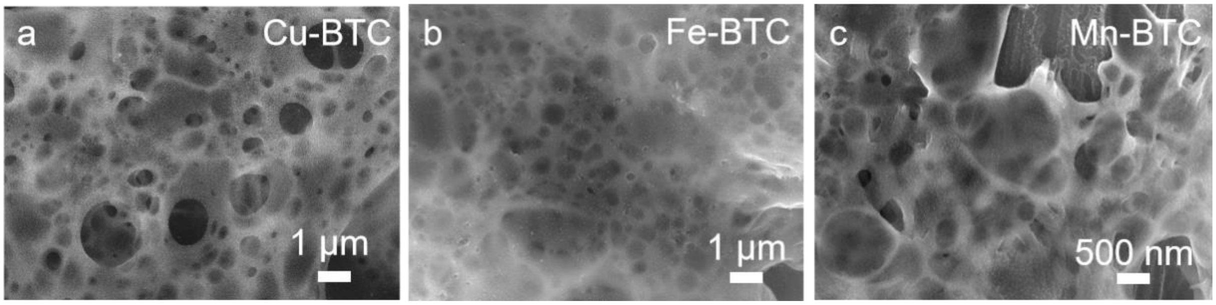

Supplementary Figure 49. SEM images of (a) Cu-BTC, (b) Fe-BTC, and (c) Mn-BTC after carbothermal shock.

Supplementary Table 1. Comparison of various CTS methods.

	1.Substrate	Special substrate?	2.Precursor	Precursor design?	Compositions	In situ assembly / tunable pores?	3. Particles	4.Hierachical porosity?	Reference
Conventional CTS	Carbon	X	Metal salt (e.g., CoCl2)	X	None specific	X	~10-20 nm	X	Science 2018, 359, 1489-1494 (our previous work)
Enhanced CTS (enhancing the metal-substrate interaction)	CO_2 activated carbon (defective carbon)	√	Metal salt (e.g., H2PtCl6)	X	None specific	X	~5 nm (high density) ~0.8 nm (low density)	X	ACS AMI 2019, 11, 29773 (our previous work)
	3D mesoporous carbon (defective, pore confinement)	√	Metal salt (e.g., RuCl2)	X	None specific	X	~ 3 nm (low density)	X (only mesopores)	Nano Lett. 2019, 19, 8, 5149-5158 (Lacet et. al.)
	Cellulose (maximized defective carbon)	√	Metal salt (e.g., CuCl2)	X	None specific	X	~35 nm (High density coverage > 85%)	X	Sci. Adv. 2021, 7, eabk2984 (Song et. al.)
Coordinated CTS (coordinated metal-ligands in precursor design)	Carbon (carbon for CTS)	X	Crystalline MOF (e.g., ZIF-67)	√	Only MOF	X	< 3 nm (high density)	X (micropores < 2 nm)	Nano Energy 2022, 97, 107125
	Carbon (carbon for CTS)	X	Metal + ligands (e.g. Co2+ & 2MeIm)	√	Coordination chemistry (Beyond MOF)	√	~ 3.2 nm (high density)	√ open/hierarchical pores (μm-nm)	**This work**

Supplementary Table 2. The heating rates control at different target heating rate.

Target heating rate (°C/s)	Current rate (A/s)	Current range (A)	Heating time (s)	Peak temperature (°C)	Actual heating rate (°C/s)
100	1	0 ~ 10	10	1063	106.3
1000	10	0 ~ 10	1	1042	1042
10000	100	0 ~ 10	0.1	1009	10090

Note: the heater size (carbon paper): length * width * thickness = 3.0 cm * 0.5 cm * 0.03 mm; Power loading mode: current output; Maximum output voltage: 30 V.

Supplementary Table 3. The peak areas (%), binding energy, and FWHM for N species in Co-N-CTS.

	**pyridinic N**	**Co-N**	**pyrrolic N**	**graphitic N**	**oxidized N**
**Binding energy (eV)**	398.22	399.02	400.25	401.1	402.16
**FWHM**	1.52	1.85	1.3	1.23	0.92
**Peak areas (%)**	33.5	27.2	14.1	19.8	5.4

Supplementary Table 4. The peak areas (%), binding energy, and FWHM for Co species in Co-N-CTS.

	**Co^0 $2p_{3/2}$**	**Co^{3+} $2p_{3/2}$**	**Co^{2+} $2p_{3/2}$**	**Satellite peaks**	**Co^0 $2p3/2$**	**Co^{3+} $2p_{3/2}$**	**Co^{2+} $2p_{3/2}$**
**Binding energy (eV)**	778.41	779.83	781.44	783.76	793.42	795.08	796.36
**FWHM**	1.33	1.99	2.37	3.5	1.86	1.63	1.63
**Peak areas (%)**	34.5	20.0	11.4	12.1	12.8	5.5	2.8

Supplementary Table 5. The peak areas (%), binding energy, and FWHM for Co species in Co-CTS.

	**Co^0 $2p_{3/2}$**	**Co^{3+} $2p_{3/2}$**	**Co^{2+} $2p_{3/2}$**	**Satellite peaks**	**Co^0 $2p_{3/2}$**	**Co^{3+} $2p_{3/2}$**	**Co^{2+} $2p_{3/2}$**	**Satellite peaks**
**Binding energy (eV)**	778.22	779.39	781.48	784.28	793.36	795.19	796.86	903.19
**FWHM**	1.16	2.33	2.72	3.5	2.02	2.17	2.95	3.5
**Peak areas (%)**	22.7	22.7	16.0	8.4	12.5	4.5	8.2	5.0

Supplementary Table 6. Performance comparison in ORR and OER between Co-N-CTS and other reported Co-based bifunctional electrocatalysts in literature.

Catalysts	ORR $E_{1/2}$ (V)	OER η_{10}(mV)	ΔE (V)	Reference
**Co-N-CTS**	0.86	280	0.65	This work
**OLC/Co-N-C**	0.86	350	0.72	Angew. Chem., Int. Ed. 2021, 60, 12759.
**NCNTFs**	0.87	370	0.73	Nat. Energy. 2016,1, 15006.
**CNT@SAC-Co/NCP**	0.87	380	0.74	Adv. Funct. Mater. 2021, 31, 2103360.
**Co_3O_{4-x} HoNPs @HPNCS**	0.83	340	0.74	Angew. Chem. Int. Ed. 2019, 58, 13840-13844.
**Co@hNCTs-800**	0.87	400	0.76	Nano Energy 2020, 71, 2211.
**Co SA/NCFs**	0.85	380	0.76	Nano Lett. 2022, 22, 2497−2505.
**Co-NCNT**	0.86	400	0.77	Energy Storage Mater. 2019, 20, 234-242.
**Co/CNWs/CNFs**	0.82	410	0.82	Adv. Funct. Mater. 2021, 31, 2105021.
**Co-POC**	0.83	470	0.87	Adv. Mater. 2019, 31, 1900592.
**CoSAs-NGST**	0.89	560	0.9	Adv. Funct. Mater. 2021, 31, 2010472.

Supplementary Table 7: The formation energy of metal with 2-MeIm.

**Ingredient**	**Ag**	**Cu**	**Co**	**Mn**	**Cr**	**Fe**	**Ni**
**Formation energy**	-0.87	-2.5	-3.44	-3.55	-4.14	-4.66	-5.8

Supplementary Table 8: The formation energy of metal with BTC.

Ingredient	Cu	Fe
**Formation energy**	-8.13	-11.55

Supplementary References

1. Yao, Y. *et al.* Carbothermal shock synthesis of high-entropy-alloy nanoparticles. *Science (80-.).* **359**, 1489–1494 (2018).
2. Yao, Y. *et al.* Ultrafast, controllable synthesis of sub-nano metallic clusters through defect engineering. *ACS Appl. Mater. Interfaces* **11**, 29773–29779 (2019).
3. Lacey, S. D. *et al.* Stable multimetallic nanoparticles for oxygen electrocatalysis. *Nano Lett.* **19**, 5149–5158 (2019).
4. Song, J.-Y. *et al.* Generation of high-density nanoparticles in the carbothermal shock method. *Sci. Adv.* **7**, eabk2984 (2021).
5. Han, Y.-C. *et al.* A general strategy for overcoming the trade-off between ultrasmall size and high loading of MOF-derived metal nanoparticles by millisecond pyrolysis. *Nano Energy* **97**, 107125 (2022).
6. Liang, Z. *et al.* Highly Curved Nanostructure-Coated Co, N-Doped Carbon Materials for Oxygen Electrocatalysis. *Angew. Chemie* **133**, 12869–12874 (2021).
7. Xia, B. Y. *et al.* A metal–organic framework-derived bifunctional oxygen electrocatalyst. *Nat. Energy* **1**, 15006 (2016).
8. Li, J. *et al.* Dual-phasic carbon with co single atoms and nanoparticles as a bifunctional oxygen electrocatalyst for rechargeable Zn–air batteries. *Adv. Funct. Mater.* **31**, 2103360 (2021).
9. Ji, D. *et al.* The kirkendall effect for engineering oxygen vacancy of hollow Co_3O_4 nanoparticles toward high-performance portable zinc–air batteries. *Angew. Chemie* **131**, 13978–13982 (2019).
10. Zhou, Q. *et al.* Template-guided synthesis of Co nanoparticles embedded in hollow nitrogen doped carbon tubes as a highly efficient catalyst for rechargeable Zn-air batteries. *Nano Energy* **71**, 104592 (2020).
11. Han, Y. *et al.* Stabilizing Cobalt Single Atoms via Flexible Carbon Membranes as Bifunctional Electrocatalysts for Binder-Free Zinc–Air Batteries. *Nano Lett.* **22**, 2497–2505 (2022).
12. Pei, Z. *et al.* Enabling highly efficient, flexible and rechargeable quasi-solid-state zn-air batteries via catalyst engineering and electrolyte functionalization. *Energy Storage Mater.* **20**, 234–242 (2019).
13. Xia, C. *et al.* Electrospinning synthesis of self-standing cobalt/nanocarbon hybrid membrane for long-life rechargeable zinc–air batteries. *Adv. Funct. Mater.* **31**, 2105021 (2021).
14. Li, B. *et al.* Framework-porphyrin-derived single-atom bifunctional oxygen electrocatalysts and their applications in Zn–air batteries. *Adv. Mater.* **31**, 1900592 (2019).
15. Ban, J. *et al.* Dual evolution in defect and morphology of single-atom dispersed carbon based oxygen electrocatalyst. *Adv. Funct. Mater.* **31**, 2010472 (2021).
